# Inhibition of CpLIP2 Lipase Hydrolytic Activity by Four Flavonols (Galangin, Kaempferol, Quercetin, Myricetin) Compared to Orlistat and Their Binding Mechanisms Studied by Quenching of Fluorescence

**DOI:** 10.3390/molecules24162888

**Published:** 2019-08-08

**Authors:** Ruba Nasri, Luc P R Bidel, Nathalie Rugani, Véronique Perrier, Frédéric Carrière, Eric Dubreucq, Christian Jay-Allemand

**Affiliations:** 1 UMR 1208 IATE, Université de Montpellier, 34095 Montpellier, France; 2 UMR 1208 IATE, Montpellier SupAgro, Place Viala, 34060 Montpellier, France; 3 UMR 1334 AGAP, INRA, Place Viala, 34060 Montpellier, France; 4 Département Bio-MV, Université de Montpellier, 34095 Montpellier, France; 5 UMR 7281 BIP, Aix Marseille Université, CNRS, 31 chemin Joseph Aiguier, 13402 Marseille cedex 09, France

**Keywords:** recombinant CpLIP2 lipase, flavonols, orlistat, fluorescence quenching, inhibition, hydroxylation, tryptophan residues, docking

## Abstract

The inhibition of recombinant CpLIP2 lipase/acyltransferase from *Candida parapsiolosis* was considered a key model for novel antifungal drug discovery and a potential therapeutic target for candidiasis. Lipases have identified recently as potent virulence factors in *C. parapsilosis* and some other yeasts. The inhibition effects of orlistat and four flavonols (galangin, kaempferol, quercetin and myricetin) characterized by an increasing degree of hydroxylation in B-ring, were investigated using ethyl oleate hydrolysis as the model reaction. Orlistat and kaempferol (14 µM) strongly inhibited CpLIP2 catalytic activity within 1 min of pre-incubation, by 90% and 80%, respectively. The relative potency of flavonols as inhibitors was: kaempferol > quercetin > myricetin > galangin. The results suggested that orlistat bound to the catalytic site while kaempferol interacted with W294 on the protein lid. A static mechanism of interactions between flavonols and CpLIP2 lipase was confirmed by fluorescence quenching analyses, indicating that the interactions were mainly driven by hydrophobic bonds and electrostatic forces. From the Lehrer equation, fractions of tryptophan accessibility to the quencher were evaluated, and a relationship with the calculated number of binding sites was suggested.

## 1. Introduction

Flavonoids are a subgroup of phenolics belonging to plant secondary metabolites. They are classified in several subclasses including chalcones, flavanones, flavonols, flavones, isoflavones, flavan-3-ols, flavan 3-4 diols, anthocyanidins and aurones, each varying in their chemical structures and properties [1]. Flavonoids are often stored in tissues under various glycoside forms, playing functional roles within the plant cells at the physiological level [2,3,4,5], but are also directly involved in signaling pathways and plants defense through multiple interactions with pathogenic microorganisms [6,7]. Flavonoids are also bioactive compounds having therapeutic and preventive effects [8] through antioxidant, anti-inflammatory, anti-mutagenic, anti-carcinogenic [9], antibacterial and antifungal activities [10,11]. A strong relationship between the degree of hydroxylation of flavonoid structures and their fungicidal activities has been reported [12]. Indeed, several studies have described flavonoids as natural alternatives to antifungal agents because of the emergence of drug-resistant strains in candidiasis [13], and candidemia [14,15]. The pharmacological and biochemical activities of flavonoids are tightly linked to their antioxidant activities [15], and their capacities to inhibit numerous enzymes such as xanthine oxidase, cyclo-oxygenase, lipoxygenase, phosphoinositide 3-kinase [16], and phospholipases [17].

For several decades, the relationships between the poly-hydroxylation of flavonoids and their inhibitory effects on a wide range of enzymes has been of great interest in research on chemical and biological functionalities. Indeed, a tight relationship was reported between the flavonoid structural requirements and specificity of inhibition of digestive enzymes α-glucosidase, α-amylase and aldose reductases [18]. An in vitro screening of 14 flavonoids (60 µM) was undertaken on phosphatidyl-inositol-3-kinase for their anti-cancer properties. The most potent inhibitor among the tested flavonols was myricetin (MYR). These results confirmed the relationship between structure and function, where the number and the position of the OH group on the B-ring, including the degree of unsaturation of the C2-C3 bond, were essential to obtaining high inhibition [19]. In addition, MYR was also identified as the strongest inhibitor among 16 main flavonoids against mammalian DNA polymerases and human topo-isomerases by in vitro investigation [20]. Strong inhibition of human secretory phospholipases by some flavonoids such as quercetin (QUE) has been reported, and the importance of hydroxy groups on the A and B rings has also been clearly confirmed [17]. This involves different types of interactions such as π-stacking, hydrophobic interactions, hydrogen bounds with water molecules or with special amino acid residues (phenylalanine (F), histidine (H), cysteine (C), lysine (K). In most cases, a high degree of hydroxylation fits with potent interaction and inhibition of enzymes. However, it appears there is no simple relationship between the polarity of the molecules due to hydroxylation and their capacity for enzyme inhibition. Indeed, kaempferol (KAE) showed the highest inhibition against *C. rugosa* and *C. albicans* lipases [21,22,23]. These results could be very informative for investigating the mechanism of action and, therefore to better understand the highest inhibitory effects of KAE and MYR among the flavonoids.

**Figure 1:**
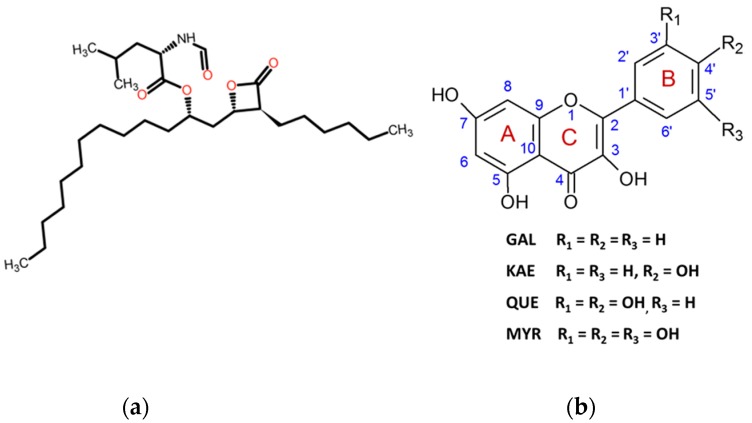
Chemical structures of (**a**) orlistat (tetrahydrolipstatin; THL) and (**b**) investigated flavonols in this study: GAL, galangin; KAE, kaempferol; QUE, quercetin; MYR, myricetin. Flavonol skeleton shows the numbering system of three rings A, B and C.

Lipases (EC 3.1.1.3) are ubiquitous enzymes that naturally catalyze the hydrolysis of triacylglycerols such as in fats and oils into free fatty acids and glycerol. The diversity of their origins (plants, animals and microorganisms) ensures not only their natural availability but also their huge variety of functional characteristics for applications in diverse sectors [24]. Fungal lipases are well studied as biotechnological agents and have found multiple industrial applications in agronomic and health fields. However far fewer studies have considered their potential as virulence factors [25]. The lipase/acyltransferase CpLIP2 from *Candida parapsilosis* (*Cp*) CBS 604 has been studied in our laboratory for more than 20 years for its high capacity to catalyze acyl transfer reactions even in aqueous media [26,27,28,29]. Recently, several studies have identified the role of secreted lipases in several strains of *Cp* as a major virulence factor which contributes to the pathogenicity of this human opportunistic fungal pathogen [30,31,32]. Additionally, other extracellular lipases of *Candida* species, such as *C. rugosa,* have been reported as key virulence factors of candidiasis, enabling yeasts to penetrate into the host cells. Thus, the discovery of novel inhibitors with high efficiency on the pathogens, but with low toxicity for human and animal cells, represents a very important stake for pharmacology and for the development of innovative therapeutic strategies. Clinical trials on healthy volunteers [33] resulted in the approved use of orlistat (Figure 1a), also known as tetrahydrolipstatin (THL), which is a well-known covalent inhibitor of lipases such as human pancreatic and gastric lipases. THL reacts with the nucleophilic serine residue of the catalytic triad of lipases [34,35,36].

To our knowledge, no published work reports such an interaction between CpLIP2 lipase and phenolics or THL molecule. The formation of complexes between phenolics and enzymatic macromolecules can be investigated by numerous analytical methods through enzymatic catalysis assays [37], micro-calorimetry, spectroscopic measurement such as fluorescence quenching, SPR [38,39] and molecular docking [40,41]. In this work, we first investigated the mode of interactions of CpLIP2 with THL and four flavonols, galangin (GAL), KAE, QUE and MYR (Figure 1b), which differ by the number of OH groups (0 to 3) on the B-ring, through three complementary approaches: enzymatic, fluorescence quenching and molecular modeling. The effects of pre-incubation time and inhibitor concentration were studied using ethyl oleate as the lipid substrate. The reaction was performed in aqueous biphasic medium with high thermodynamic activity of water (a_w_ > 0.95) with 50 mM sodium phosphate buffer at pH 6.5 and 1% ethanol. Fatty acid ethyl ester hydrolysis was followed by GC analyses. Then, the quenching of CpLIP2 intrinsic tryptophan (W) fluorescence was studied using the Stern-Volmer theory. Correlations with the protein structure of CpLIP2 and parameters such as the solvent accessibility area of tryptophan residues were estimated by molecular docking and structural modelling.

## 2. Results

### 2.1. THL Inhibits Rapidly and Strongly the Hydrolytic Activity of CpLIP2

**Figure 2:**
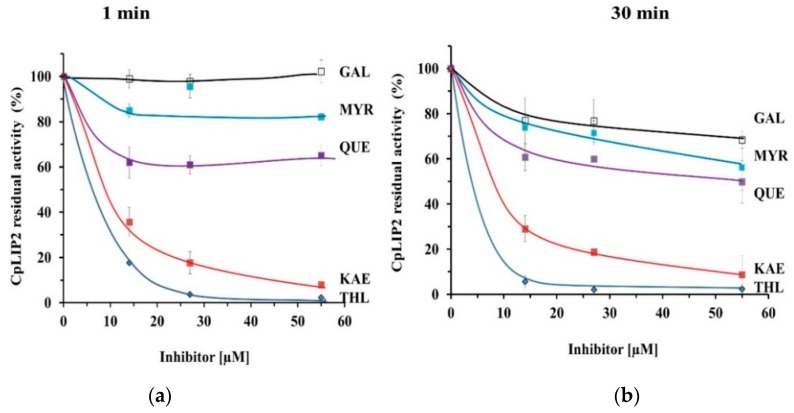
Inhibition of CpLIP2 hydrolytic activity by THL (positive control), and four flavonols (GAL, KAE, QUE, MYR) tested at various concentrations (14, 27.5, 55 µM equal to 25, 50, 100-fold the final concentration of CpLIP2 lipase at 0.55 µM, respectively). Each potential inhibitor was pre-incubated with CpLIP2 at 25 °C with continuous stirring in presence of ethanol 1% for 1 min (**a**) and 30 min (**b**) of pre-incubation time. Hydrolysis reactions of ethyl oleate (EO) were conducted at 30 °C for 15 min, and then measured by gas chromatography as described in the Section 4. Results are expressed as mean values ± SD, n = 6–9. THL, orlistat; GAL, galangin; KAE, kaempferol; QUE, quercetin; MYR, myricetin.

A strong inhibition of the hydrolysis activity of CpLIP2 was observed after only 1 min of pre-incubation for all THL concentrations compared to the control. In the presence of the lowest THL concentration (14 µM), corresponding to a 25-fold molar excess compared to CpLIP2, the residual activity was only 17.7% ± 0.9 (Figure 2). Under the same conditions, the residual activity after 1 min was 3.9% ± 0.5 and 2.3% ± 0.1, respectively, in the presence of 27.5 and 55 µM of THL (Figure 2). The inhibition was more pronounced after 30 min of pre-incubation. Considering the strong inhibition observed with THL, this compound was then considered as a positive control for inhibition tests.

### 2.2. KAE Is the Strongest Inhibitor among the Flavonols Tested

KAE has a potent inhibitory effect very similar to THL. As shown in Figure 2, THL was the strongest inhibitor against CpLIP2, at 50-fold the lipase concentration (27.5 µM), with less than 2% residual activity after 1 min of pre-incubation. KAE was the only flavonol tested to inhibit the activity of CpLIP2 by more than 80% at this concentration. GAL, which is not hydroxylated on its B-ring, had the lowest inhibitory effect, that could be detected only after 30 min of pre-incubation. 

The effect of hydroxylation degree and OH position on the B-ring was investigated. The inhibition of CpLIP2 lipase by single flavonols showed a strong relationship with differences in the chemical structures, apparently depending on the degree of hydroxylation on B-ring. However, the absence of OH group in 4′-position on the B-ring (GAL) limited drastically the inhibition, even giving no inhibition 1 min after pre-incubation (Figure 2), and exhibiting the lowest inhibition compared to the other flavonols in all cases. KAE, with a single hydroxy group in 4′-position, showed the strongest inhibitory rate reaching 92% at molar excess of 100-fold CpLIP2, and was dose-dependent. It induced the maximal inhibition rate for the shortest pre-incubation time and showed a relatively stable effect irrespective of the pre-incubation time tested. For QUE, inhibition was 37%, 39% and 35% at 14, 27.5 and 55 µM, respectively. Similar to QUE, MYR inhibited CpLIP2 lipase activity by about 20% only. Thus, the differences between these different complexes (ligands-lipase) involving particularly KAE or THL might reflect two different mechanisms of inhibition that have been investigated by fluorescence and molecular docking approaches.

The effect of inhibitor concentrations on the hydrolysis reaction rate in standard conditions was assumed to follow either a non-competitive or an uncompetitive model, corresponding to Equation (1):

(1) $$K_{i}=\frac{I}{\frac{{\;V}_{0}}{V_{i}}-1}$$

where V_0_ and V_i_ are the initial rates of the reaction without or with inhibitor at concentration I.

The Ki values estimated after 1 and 30 min of pre-incubation were 1.2–0.6 µM for THL and 4–6 µM for KAE, respectively. This confirmed that extending the pre-incubation time from 1 to 30 min had only a limited impact on the inhibition. For the other compounds, the K_i_ values estimated from data after 30 min of pre-incubation were 60 µM for MYR, 80 µM for QUE and >150 µM for GAL. This showed that inhibition by KAE was in the same order of magnitude as by THL, although with a 5–10 times higher K_i_ value, whereas GAL had a limited effect. The inhibitory effect of QUE and MYR was about 100 times lower than that of THL.

### 2.3. Interactions between CpLIP2 Lipase and 4 Flavonols by Means of Fluorescence Spectroscopy 

The amino acids sequence of CpLIP2 lipase contains seven tryptophan (W) residues, which contribute to its hydrophobicity and fluorescence properties. The position of each W residue in the protein sequence is: 51, 177, 188, 294, 347, 350 and 379. The emission of fluorescence of CpLIP2 lipase at 350 nm after excitation at 285 nm is mainly explained by its seven W residues. The concentration of CpLIP2 lipase (2.5 µM) was determined in order to detect significant fluorescence in a neutral buffer solution containing 0.1% (17 mM) ethanol. As both tyrosine (Y) and W residues could be excited intensively at 280 nm, the excitation beam of 285 nm was chosen to minimize the Y fluorescence contribution. Then, we determined the increased ionic strength by adding 300 mM, NaCl leading to a higher fluorescence intensity that could indicate a possible change of the conformation of the protein (Figure S2, Supplementary Materials). CpLIP2 showed its maximum emission at 352 nm, after being excited at 285 nm as seen in Figure S3 (Supplementary Materials). A red shift of 2 nm was observed from the maximum of fluorescence intensity of native free lipase at 352 nm moving into denatured lipase (unfolded protein) at 354 nm. The lipase was unfolded by incubation at 100 °C, which exposed its W residues to the solvent. Changes affecting W residues, surrounded by a more or less hydrophilic microenvironment, modify their fluorescence intensity [37]. Such exposition could explain the lowering of fluorescence intensity upon the unfolding of the CpLIP2 lipase. The fluorescence emission spectrum (excitation wavelength 285 nm) of native CpLIP2 lipase was also investigated after a pre-incubation step with a 20-fold molar excess THL at 25 °C for 30 min. The results showed a blue shift of 4 nm when the native free lipase was inhibited (Figure S3, Supplementary Materials). This could indicate a less polar microenvironment of W residues [38].

#### 2.3.1. Analysis of CpLIP2 Fluorescence Quenching by Flavonols

**Figure 3:**
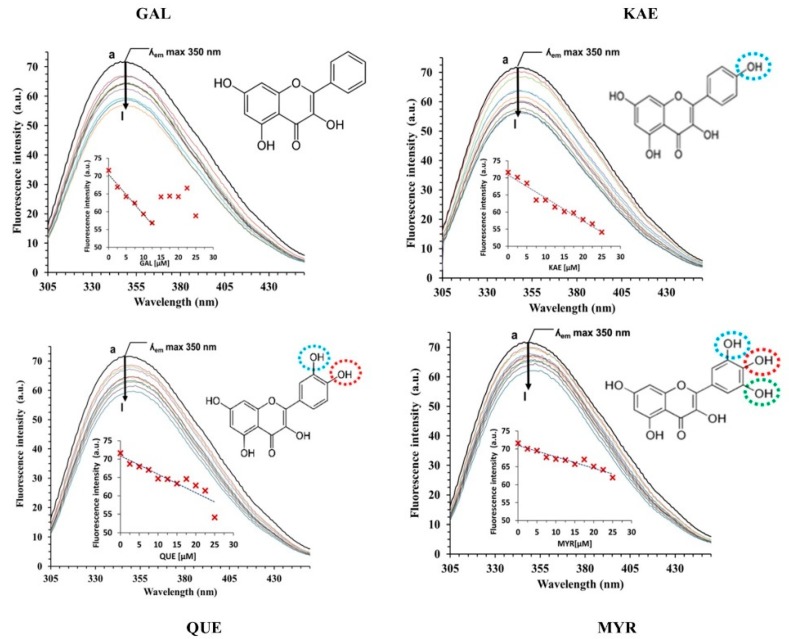
Fluorescence emission spectra of CpLIP2 lipase alone or with increasing concentrations of single flavonols (coloured lignes). The concentration of CpLIP2 was 2.5 µM, and the final concentrations of flavonols correspond to the following gradient from a to l (see arrow): 0, 2.5, 5.0, 7.5, 10.0, 12.5, 15.0,17.5, 20.0, 22.5 and 25 µM of GAL (galangin), KAE (kaempferol), QUE (quercetin), and MYR (myricetin). Measurements at 293 K, pH: 7.0, 300 mM NaCl; EtOH 0.1%. The excitation wavelength was 285 nm, and the emission spectra was recorded in the wavelength range of 305–450 nm. Increment slits 1 nm. Corrections of inner filter was applied to all spectra. The four insert plots represent the fluorescence intensities at ʎ_em_ = 350 nm as a function of quencher concentrations.

The fluorescence quenching spectra of CpLIP2 lipase submitted to increasing concentrations of GAL, KAE, QUE and MYR are shown in Figure 3. A remarkable quenching of the CpLIP2 fluorescence emission was observed. The strongest effect was observed with KAE while the lowest was with GAL. The maximum wavelengths of fluorescence emission of CpLIP2 lipase showed a slight blue shift in all cases. These results suggest that GAL, KAE, QUE and MYR interact with CpLIP2, modifying the microenvironment of one or more indole rings from the accessible W residues. The fluorescence quenching is usually described [37] by the linear Stern–Volmer Equations (2) and (3):

(2) $$\frac{F_{0}}{F}\;=\;1\;+{\;K}_{\mathrm{sv}}\;\left[Q\right]\;=\;1\;+{\;K}_{q}\tau_{0\;}\left[Q\right]$$

(3) $$K_{q}=\frac{K_{\mathrm{sv}}}{\tau_{0}}$$

where F_0_ and F are the intrinsic fluorescence intensities of CpLIP2 lipase (fluorophore) in absence or presence of quencher (flavonol), respectively. K_q_ is the bimolecular quenching constant, τ_0_ is the lifetime of the fluorophore in the absence of the quencher, [Q] is the concentration of the quencher, and K_SV_ is the Stern–Volmer quenching constant.

Linear Stern–Volmer plots indicate that a single class of fluorophores exists in the protein, and that only one mechanism (dynamic or static) of quenching occurs. When the Stern–Volmer plots show an upward curvature towards the y-axis, a combined mechanism between dynamic and static quenching can occur [37].

The mechanism of quenching, occurring between CpLIP2 lipase and flavonols, was characterized by determining the values of K_q_ and the thermodynamic parameters by varying the temperature. Hence, Equation (2) was applied to determine K_SV_ by linear regression of a plot of F_0_/F against [Q]. In both cases, the bimolecular quenching constant K_q_ can be calculated by the ratio between K_SV_ and τ_0_, itself determined at 1.59 ns for Human Pancreatic Lipase [39].

When the K_q_ value is much greater than >2.0 × 10^10^ L mol^−1^ s^−1^ (maximum value for scatter collision quenching constant), the fluorescence quenching process follows a static quenching model. In our study, as seen in Table 1 for GAL, KAE, QUE and MYR, the order of magnitude of the binding constant K_q_ was estimated to be 10^13^. We can thus deduce that a complex may have formed between CpLIP2 lipase and the four tested flavonols, and that the main mechanism of quenching followed a static model in all cases. By increasing the temperature, the phenomenon of diffusion increases, which can cause more dissociation of weaker bounds, decrease the stability of the complex in the case of the static mechanism [40] and increase the collisional diffusion for the dynamic mechanism. As a result, a lower value of bimolecular quenching constants K_q_ is expected for the static mechanism and a higher value for the dynamic mechanism with higher temperatures [37].

Aiming to confirm our results about the mechanism of quenching, each flavonol-CpLIP2 mixture was incubated at 298, 308, 318, and 328 K. The fluorescence quenching data were analyzed by Stern–Volmer plots using Equation (2). Data listed in Table 1 show that the Stern–Volmer constant K_sv_ for the four flavonols clearly decreased when the temperature increased, showing a clear negative correlation with temperature. These results suggest that GAL, KAE, QUE, and MYR are responsible for the quenching of CpLIP2 fluorescence through a static quenching mechanism. Thus, each flavonol interacts with CpLIP2 lipase by a binding mechanism that has to be identified. Note that the linearity of the plot F_0_/F against [Q] confirms that there is only a single population of fluorophore in the protein that interacts similarly with the quencher indicating a unique mechanism of quenching [41].

**Figure 4:**
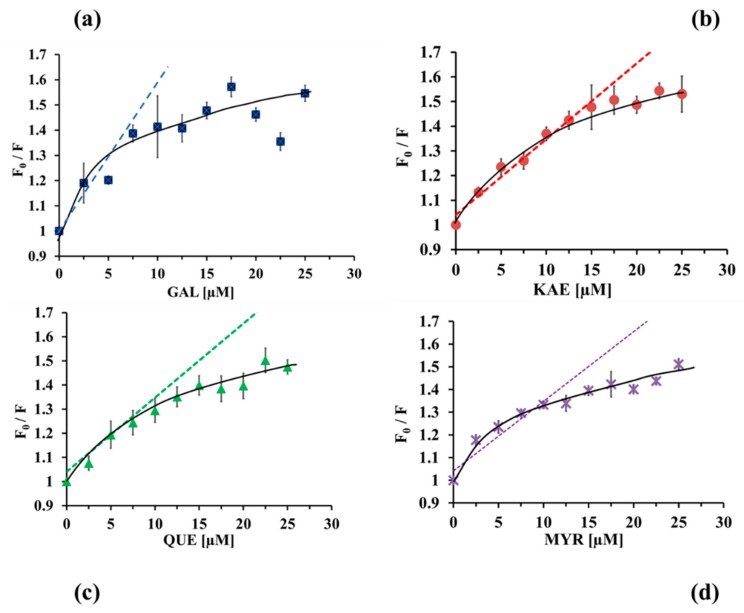
The Stern–Volmer plots for fluorescence quenching by (**a**) GAL (galangin), (**b**) KAE (kaempferol), (**c**) QUE (quercetin), (**d**) MYR (myricetin) at T = 298 K. CpLIP2 lipase concentration is 2.5 µM, pH 7.0. The fluorescence emission intensity was recorded at ʎ_ex_ = 285 nm and ʎ_em_ = 350 nm. F_0_/F versus [Q] plots exhibiting negative deviation from the linearity towards the x-axis. Bars indicate the SD with n = 9. (**a**–**d**) the final concentrations of flavonols correspond to the following gradient from 0, 2.5, 5.0, 7.5, 10.0, 12.5, 15.0,17.5, 20.0, 22.5 and 25 µM. The trend lines represent Stern–Volmer quenching when all W are equally accessible to flavonols. The up-down curvatures towards the x-axis represent Lehrer model taking into account the inaccessibility of some W of CpLIP2 lipase.

Data calculated according to the Stern–Volmer theory (Figure 4) showed an up-down curvature towards the x-axis, which could indicate the presence of two populations of W residues where one of them is not accessible to the quencher. The inaccessible or buried residues are responsible for the remaining fluorescence, which is probably independent of the quencher concentration [37]. In our study, the linear Stern–Volmer plot was confirmed only for weak concentrations (2.5–15 µM), whilst with higher concentrations (15–25 µM), F_0_/F versus [Q] plots exhibited a negative deviation from the linearity towards the x-axis. An up-down curvature towards the x-axis has been shown for the four flavonols (Figure 4), indicating the presence of two populations of fluorophores, part of which would not be accessible. That means that some W residues are buried inside the lipase structure and that other residues are exposed to the solvent. Therefore, data were analyzed through the Lehrer equation, also known as the modified (or non-linear) Stern–Volmer Equation (4):

(4) $$\frac{{\;F}_{0}}{{\;F}_{0}-F\;}=\;\frac{1}{\;f_{a}\;K_{\mathrm{sv}}\;}\frac{1}{\;\left[Q\right]\;}+\frac{1}{\;f_{a}\;}$$

F_0_ and F are the intrinsic fluorescence intensities of CpLIP2 lipase in the absence or presence of quencher (flavonol), respectively, [Q] is the concentration of the quencher, *f_a_* is the fraction of accessible fluorophores, and K_SV_ is the effective Stern–Volmer quenching constant for the accessible W residues (fluorophores). As listed in Table 1, *f_a_* values varied from 0.25 to 0.69, depending on the structure of flavonols and temperature. The highest accessibility of W residues was 0.69% for QUE at the highest temperature 328 K, while the factor of accessibility *f_a_* for KAE, MYR, GAL decreased in the same order. The lowest accessibility was 0.25 at 328 K for GAL. The magnitude of order K_sv_ of the Stern–Volmer quenching constant of the exposed W residues was estimated 0.5 to 4.2 × 10^5^ L·mol^−1^, which suggested a relatively high affinity between flavonols and CpLIP2 lipase.

#### 2.3.2. Binding Constants and Number of Binding Sites

For static quenching, the equilibrium between free and bound CpLIP2 lipase, when the ligand molecules bind independently to equivalent sites of macromolecules, can be described by the following Equation (5):

(5) $$\log_{10}\frac{F_{0}-F}{F}=n\log_{10}\left[Q\right]+\log_{10}K_{a}$$

where K_a_ is the binding constant and n the number of binding sites per CpLIP2 molecule.

The results were determined for all flavonols tested at different temperatures from the plots of log_10_ F_0_ − F/F versus log_10_ [Q] and listed in Table 2. The plots show a linear relationship from which the slope equal to n and intercept on y-axis equal to log_10_ K_a_. Binding modes between the four flavonols and CpLIP2 lipase were determined via Stern–Volmer plots and summarized in Table 2. For the four flavonols, the K_a_ values increased when the temperatures increased. In addition, the n values were less than 1.0 in all cases and increased according to the temperature. The highest values were obtained with QUE (0.78–0.81), whilst the lowest values were observed with MYR (0.43–0.44). Values of the dissociation constant of complexes were deduced from the Stern–Volmer Equation (6) and (7), for which K_d_ is expressed as the inverse of *f_a_* K_sv_ product.

(6) $$\frac{F_{0}}{{\;F}_{0}-F}={\;K}_{d}\;\frac{1}{\;\left[Q\right]\;}+1$$

(7) $$K_{d}=\;\frac{1}{f_{a}\;K_{\mathrm{sv}}\;}$$

#### 2.3.3. Determination of Thermodynamic Parameters and the Nature of the Binding Forces 

Thermodynamic parameters were calculated to determine the type of binding forces that contribute in the CpLIP2 lipase–flavonol interactions. Change of enthalpy ΔH is the result of binding process occurring between the flavonols and the CpLIP2 lipase, mainly due to non-covalent interactions directed by Van der Waals forces, hydrogen bonds, hydrophobic interactions and electrostatic forces as previously suggested [42]. Both the enthalpy (ΔH) and entropy changes (ΔS) can be estimated from the following van′t Hoff Equation (8):

(8) $${\mathrm{Ln}\;K}_{a}\;=\;-\;\frac{\Delta H\;}{\mathrm{RT}\;}+\frac{\;\Delta S\;}{R}$$

K_a_ is the associative binding constant determined at different temperatures (T) expressed in Kelvin (298, 308, 318, and 328). R = 8.314472 × 10^−3^ kJ·mol^−1^ K^−1^ is the gas constant. From the van′t Hoff plots, the values of (ΔH) and (ΔS) were deduced from the slope and the intercept of the linear relationship between Ln K_a_ versus the reciprocal of absolute temperature 1/T.

The values of Gibbs binding free energy change (ΔG) are calculated from the following Equation (9):

(9) ΔG= ΔH − TΔS

The calculated values of ΔG are negative, which confirms spontaneous binding processes in all cases. When both enthalpy (ΔH) and entropy (ΔS) values are positive, the main interactions result in hydrophobic forces [43]. Moreover, when ΔH ~ 0, and ΔS > 0, electrostatic interactions are involved in binding processes [44]. Our data showed in Table 2, confirmed that the binding between CpLIP2 lipase and the four flavonols occurred mainly by both hydrophobic and electrostatic interactions.

#### 2.3.4. Quenching of CpLIP2 Lipase Fluorescence by KAE Mixed with THL

**Figure 5:**
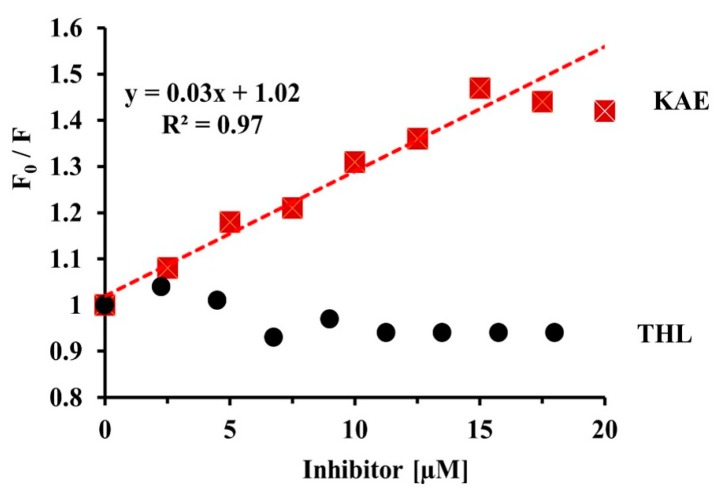
Stern–Volmer plots (F_0_/F) for quenhing of CpLIP2 lipase fluorescence by THL (black circle) and KAE (red square) as a function of their concentrations. THL concentrations: 0, 2.25, 4.5, 6.75, 9, 11.25, 13.5, 15.75, 18 µM. KAE concentrations: 0, 2.5, 5, 7.5, 10, 12.5, 15, 17.5 µM. CpLIP2 was used at 2.5 µM and inhibited by THL or KAE through a pre-incubation step for 30 min at 25 °C with 50 mM sodium phosphate buffer (pH 7.0), 300 mM NaCl and EtOH 0.01%. The fluorescence emission intensity was recorded at ʎ_ex_ = 285 nm and ʎ_em_ = 350 nm. Values represent the mean of three replicates. Correction of the inner filter was applied systematically.

As mentioned in Figure S3 (Supplementary Materials), an increase of fluorescence intensity was measured when THL used at 3-fold molar excess was incubated with CpLIP2 lipase. Then, the fluorescence intensity was stable (data not shown). By contrast, KAE was identified as a quencher of CpLIP2 (Figure 5). The complex formed between CpLIP2 lipase and KAE was confirmed by a gradual and linear increasing of the ratio F_0_/F (R^2^ = 0.97) and followed a static mechanism of quenching for the relatively low concentrations.

**Figure 6:**
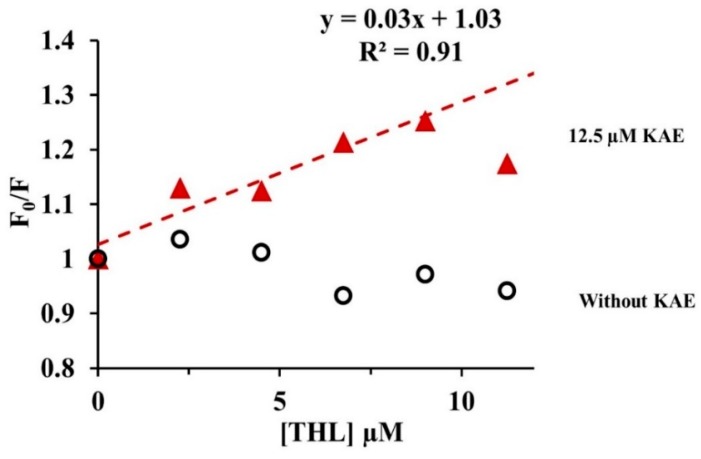
Stern–Volmer plots (F_0_/F) for quenhing of CpLIP2 lipase fluorescence as a function of THL concentration in the presence and absence of 12.5 µM KAE. CpLIP2 added at 2.5 µM and inhibited by THL concentrations: 0, 2.25, 4.5, 6.75, 9, 11.25 µM through pre-incubation period for 30 min at 25 °C, with 50 mM sodium phosphate buffer (pH 7.0), 300 mM NaCl and EtOH 0.01%. The fluorescence emission intensity was recorded at ʎ_ex_ = 285 nm and ʎ_em_ = 350 nm. Values represent the mean of three replicates. Correction of inner filter was applied systematically.

Interestingly, a remarkable quenching of fluorescence was recorded by adding 12.5 µM KAE when CpLIP2 lipase was inhibited by THL (Figure 6). The data analysis by Equation (2) governed by the Stern–Volmer theory, showed a linear Stern–Volmer plot (R^2^ = 0.91) with KAE compared to THL alone. Therefore, two different behaviors were exhibited since KAE maintains its effect as quencher, even when THL binds to lipase as competitive inhibitor. We can conclude that these two potent inhibitors of CpLIP2 seem to occupy different sites of the enzyme, leading to two different mechanisms of interaction/inhibition.

### 2.4. Energy Transfer between CpLIP2 and Flavonols

**Figure 7:**
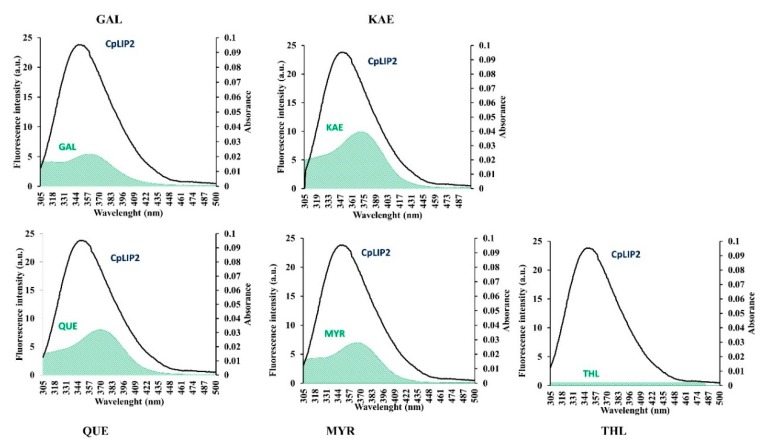
The overlap of the fluorescence emission spectrum of CpLIP2 lipase (black) with the absorption spectrum (green area) of GAL (galangin), KAE (kaempferol); QUE (quercetin); MYR (myricetin); THL (orlisat) used at 2.5 µM, T = 308 K, and pH 7.0. λ_ex_ = 285 nm, λ_em_ = 305–500 nm.

Fluorescence emission spectra of CpLIP2 lipase overlap the UV absorption spectrum of each flavonol studied, that may induce fluorescence resonance energy transfer (FRET) governed by the binding distance r between the protein behaving as energy donor and the flavonol energy acceptor. The binding distance r can be calculated via Förster theory [38] with the following Equations (10) and (11):

(10) $$E=1-\frac{F}{{\;F}_{0}}=\frac{R_{0}^{6}}{{\;R}_{0}^{6}+r^{6\;}}$$

where E is the energy transfer efficiency, which is the photons fraction absorbed and transferred by the donor to the acceptor, F and F_0_ are the fluorescence intensities of donor (CpLIP2) in the presence or absence of acceptor (flavonol) at the same concentrations. The parameter r is the distance between acceptor and donor, and R_0_ is the Förster distance, which is the critical energy transfer distance when 50% of the excitation energy is transferred from the donor to the acceptor.

(11) $$R_{0}^{6}=\;8.79\times 10^{-5\;}k^{2}N^{-4}\Phi \;J$$

where k^2^ is the spatial orientation factor of the transition dipoles from the donor to acceptor, k^2^ = 2/3 for random orientation in a fluid solution. N is the refractive index, in water solution, estimated to be equal to N = 1.336. Φ is the fluorescence quantum yield of W residues of the donor (CpLIP2). The estimated value used was Φ = 0.118. λ is the integral area of the overlap between the fluorescence spectrum of CpLIP2 lipase and the ultraviolet absorption spectrum of the flavonol acceptor (Figure 7), which was calculated by the following Equation (12):

(12) $$J=\;\frac{\sum F\left(\lambda \right)\varepsilon \left(\lambda \right)\lambda^{4}\Delta \lambda \;}{\sum F\left(\lambda \right)\Delta \lambda \;}$$

where F (λ) is the corrected fluorescence intensity of the donor (CpLIP2) from λ to Δλ, ε (λ) is the molar coefficient of absorptivity at λ. Δλ is the wavelength increment (1 nm). The value of R_0_ ranges from 20–90 Å for biological macromolecules. The calculated Förster distances between each flavonol–CpLIP2 lipase fills within the mentioned range.

The average distance r fitted within the scale of 1.2 R0 < r < 1.6 R0 for the four studied flavonols (Table 3), which indicates a high energy transfer efficiency and the non-radiative energy transfer from CpLIP2 lipase to GAL, MYR, KAE and QUE. Note that the value of r must be within this range 0.5 R0 < r < 2 R0 for indicating an effective transfer [42]. GAL and MYR showed the highest percentage of transfer efficiency, 24% and 23%, respectively, while the lowest values (12% and 6%) of transfer have been determined for KAE and QUE, respectively. According to our results, no FRET occurred between THL and CpLIP2 lipase 

### 2.5. Relationship between Binding Sites Number and Factors of W Accessibility to Flavonols

**Figure 8:**
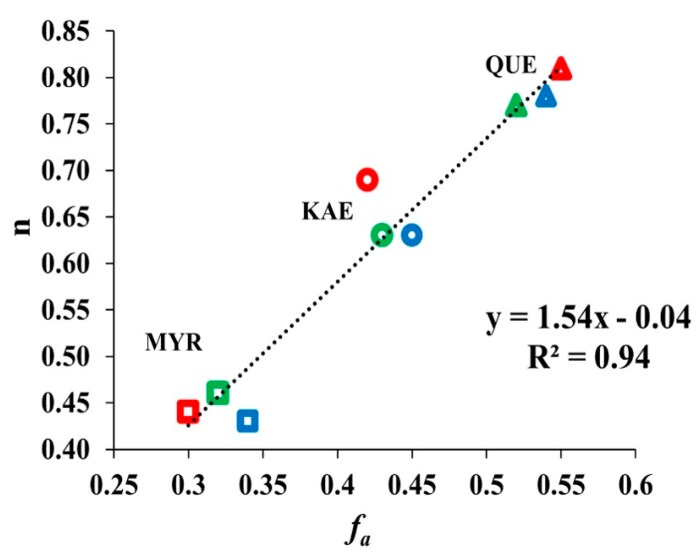
Relationship between the W accessible factor (*f_a_*) and the binding sites (n) of the three flavonols recorded at three temperatures: 298 K (blue), 308 K (green), 318 K (red). KAE, kaempferol; QUE, quercetin; MYR, myricetin represented by a circle, triangle and square, respectively.

A high correlation (R^2^ = 0.94) was found between the number of binding sites n and *f_a_* for the three flavonols tested (Figure 8) whatever the temperature used. The three hydroxylated flavonols on their B-ring are well fitted in the linear regression. KAE shows a remarkable intermediate position whereas QUE has the highest values (n = 0.76, *fa* = 0.55) and MYR the lowest values (n = 0.43, *fa* = 0.33). GAL without OH group deviated strongly from the linearity (data not shown).

As previously seen, we can hypothesize that the main attractive forces established between the CpLIP2 lipase and the flavonols tested are based on hydrophobic interactions, involving amino acid residues such as W. Such interactions could be easily explained by the log P of potential inhibitors, e.g., their polarity, which is clearly depending on their number of OH groups. But our results did not show any simple relationship. One of the questions to be addressed is: why MYR showed lower ability to bind the protein whatever the temperature used and induced week inhibition of the CpLIP2 lipase. A possible explanation is that MYR has the lowest pK_a1_ [45,46] among the inhibitors tested (pK_a1_ = 6.6) and then can easily be deprotonated at pH 6.5, thus exhibiting an ionization capacity for promoting electrostatic forces that could be responsible for preventing any interactions as well. By contrast, the pK _a1_ value of KAE is 8.2, so more stable at pH 6.5, keeping its hydrophobicity.

### 2.6. Accessible Surface Area (ASA) to Solvent of the Seven W Residues of CpLIP2 

**Figure 9:**
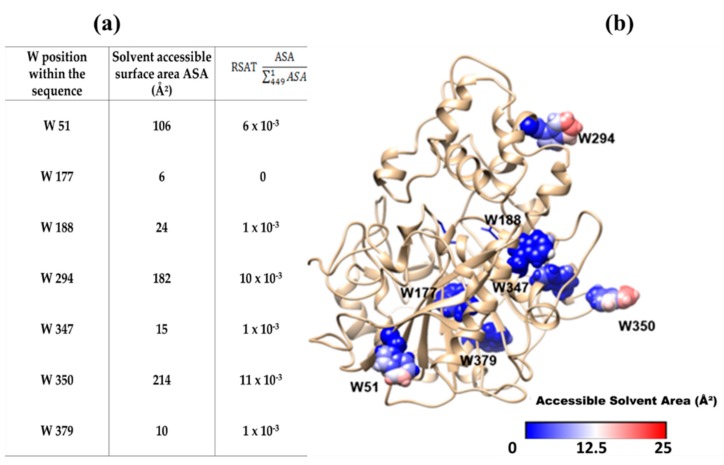
(**a**) The accessibility of the seven-W residues of CpLIP2 to solvent. Accessible solvent area (ASA) is the measure of residue solvent exposure. Total ASA (Σ^1^_449_ *ASA* = 18825 Å^2^) for CpLIP2 is the total possible solvent accessible surface area for the protein residues. RSTA is the relative accessible surface area of a protein residue to the total accessibility. (**b**) CpLIP2 3D model showing the 7 W residues colored according to their ASA. Buried W are in blue, exposed W in red, partially accessible in white.

Since the total ASA of CpLIP2 lipase was assessed Σ^1^_449_ *ASA* = 18825 Å^2^, it was possible to determine the relative solvent accessibility of the different protein residues (RSAT). The values have been determined for the 449 amino acids constituting the CpLIP2 lipase sequence. Among the 7 W residues, three of them exhibited a higher surface accessibility to solvent and may thus be more accessible to the quenchers (Figure 9a). The calculated values of W51, W294, and W350 were 106, 182, and 214 Å^2^, respectively. By contrast W177, showing the lowest values (6 Å^2^), is located in a buried position within the hydrophobic area of the protein, with a greater capacity for emitting fluorescence than the other W residues. Thus, the major part of the protein fluorescence detected may be mainly associated to W177, W188, W347 and W379.

Moreover, the relatively weak variations of fluorescence due to the quenching phenomenon, from which the relevant theory of Stern–Volmer has been developed, can be attributed mainly to the three W residues located in relative high accessible surface areas, as mentioned above. Interestingly, the W294 residue shows the highest ASA. It belongs to the area involved in the CpLIP2 catalytic activity (Figure 9b). The accessible area, containing W294, is a zone where flavonols and THL can establish non-covalent bounds to the protein surface with the highest probability.

### 2.7. Relationship between Quenching Constant (K_q_) and Hydrolytic Activity of CpLIP2 Lipase when Inhibited by Hydroxylated Flavonols on Their B-Rings

**Figure 10:**
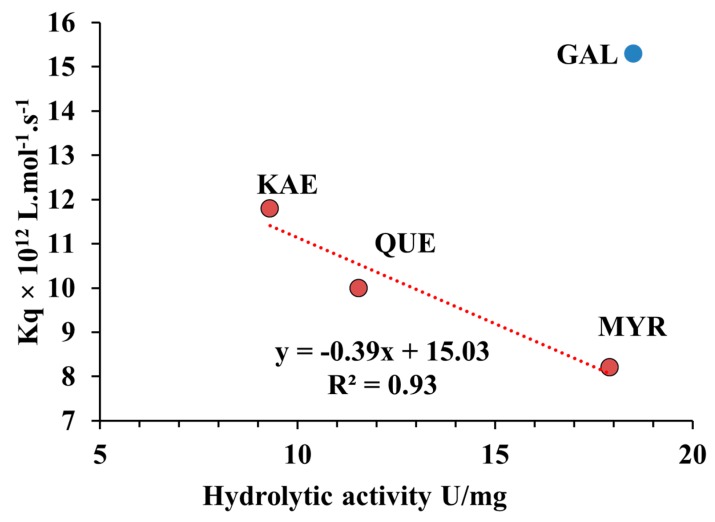
Relationship between the hydrolytic activity of 2.5 µM CpLIP2 lipase measured at 30 °C mixed with each flavonol (GAL, KAE, QUE, MYR) at 14 µM after 30 min of pre-incubation and their bimolecular quenching constant K_q_ determined at 35 °C. GAL, galangin, KAE, kaempferol; QUE, quercetin; MYR, myricetin.

A high correlation (R^2^ = 0.93) was found between the inhibitory effect on CpLIP2 hydrolytic activity and the values of the quenching constant (K_q_) for the three hydroxylated flavonols tested at 14 µM after 30 min of pre-incubation (Figure 10). The linear regression equation was K_q_ = −0.39x + 15.03 indicating that K_q_ is inversely correlated with the inhibition effect induced by flavonols, respecting the order of hydroxylation degree (Figure 10). KAE, which revealed the strongest inhibition effect on the CpLIP2 lipase activity, has the highest value of K_q_ constant mainly depending on the ratio F_0_/F evolution according to the concentration of quenchers. GAL was not fitted in the linear regression. These results could suggest that the level of inhibition on CpLIP2 lipase would be evaluated directly by fluorescence through the bimolecular quenching constant, but only if phenolics have similar properties (log P) in the solvent conditions used. Moreover, no correlation was found between the inhibitory effect on CpLIP2 hydrolytic activity and *f_a_* for the 4 flavonols tested (data not shown).

## 3. Discussion

### 3.1. THL and KAE Are Potent Inhibitors of CpLIP2 Lipase, with Different Complexation Mechanisms 

**Figure 11:**
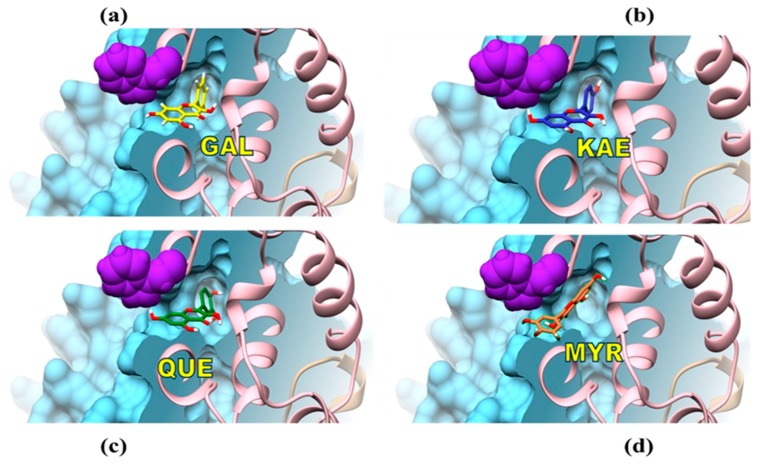
Predicted binding mode between the four flavonols (GAL, KAE, QUE, MYR) and the CpLIP2 lipase achieved by docking experiments. Surface representation of the 3D structure of CpLIP2 lipase showed in bleu cyan color indicating the major potential binding sites of the flavonols adjacent to W294 showed by violet cluster. α-helixes are showed in pink golden. The suggested binding model occupied the access to the catalytic site of CpLIP2 lipase. (**a**) GAL, galangin; (**b**) KAE, kaempferol; (**c**) QUE, quercetin; (**d**) MYR, myricetin with their own binding energy calculated at −9.1, −8.6, −8.6, −8.1 kcal/mol, respectively.

The inhibitory effect provided by THL on the hydrolytic activity of CpLIP2 lipase was faster and stronger relative to the effect of KAE, itself showing the highest inhibition among the four flavonols tested. Inhibition by THL occurred at the lowest concentration (10-fold less than the KAE) and after only a short period of pre-incubation (1 min), reaching almost 100% of inhibition with high stability. Furthermore, THL binding to CpLIP2 resulted to a 4 nm blue shift of protein fluorescence, indicating conformational changes of the protein, which did not occur with the four flavonols. Fluorescence intensity at 350 nm increased with THL binding (Figure S3, Supplementary Materials) whereas it decreased with KAE and other flavonols according to Stern–Volmer quenching. The FRET phenomenon was observed with KAE, but not with THL. The fluorescence quenching of CpLIP2 by KAE also occurred when it was previously inhibited by THL, suggesting that the two ligands did not overlap in the same sites. Indeed, docking simulations revealed that THL was probably located in the active site whereas KAE was bound to lipase surface. Taken all together, these results bring reliable arguments that THL and KAE have different binding modes, conferring different inhibition mechanisms of hydrolytic activity. This efficient inhibition for CpLIP2 lipase is in good accordance with previous results [36], where 100-fold molar excess of THL was incubated during few minutes with a human pancreatic lipase (HPL) at 25 °C. A reversible inhibition by the formation of a covalent complex between THL open β-lactone ring with the HPL catalytic serine was identified. In addition, a recent investigation reported the in vitro and in silico inhibitory effect of THL against *C. rugosa* lipase (CRL), aiming to develop a suitable treatment of candidiasis by targeting CRL activity. THL was defined as a competitive inhibitor that can establish both hydrophobic and polar interactions with CRL [23,47]. The effect of THL on CPLIP2 lipase, investigated by fluorescence spectroscopy, showed an expected increasing of fluorescence spectrum from native CpLIP2 after 30 min of incubation with the inhibitor. This result suggests that large conformational changes occurred, due to occupation of the active site by THL. Consequently, notable changes would occur in the microenvironment of W294, which is one of the most exposed W residues and localized near the active site, with SAS value close to 182 Å^2^. Undoubtedly, W294 changed its exposure to the solvent and established new hydrophobic bonds with the phenolic inhibitor. Our results confirmed those published by Lüthi-Peng and Winkler [35], in which the increasing of fluorescence was linked to an acylation of HPL by THL, inducing covalent bonding in the active site and conformational changes of HPL. Although we have no direct evidence that THL binds to the catalytic site of CpLIP2 lipase, the fluorescence changes combined with enzymatic inhibition data indicate that such an interaction is likely. Surprisingly, KAE exhibited also a relatively strong and direct inhibitory effect, comparable to the THL effect on CpLIP2 hydrolytic activity after 1 min of pre-incubation, showing also a dose-dependent inhibition mainly expressed during the first 30 min of the incubation period. A similar dose-dependent reduction of extracellular lipase activity has been reported when KAE or apigenin were added in the reaction mixture [22]. Moreover, natural inhibitors with low toxicity have been explored for preventive and therapeutic purposes of lipase-related infections [21]. The authors of this study reported the highest inhibitory effect against *C. rugosa* commercial lipase by KAE or (±)-catechin with IC50 of 6.3 mM and 7.5 mM, respectively. Indeed, KAE showed reliable inhibitory effects against *C. rugosa* and *C. albicans* lipases [21,22]. A recent report described a possible covalent bound created between a gastric lipase and the rhamnosylated myricetin [48]. Molecular docking modeling indicated that the hydroxy group of the catalytic Ser153 residue of gastric lipase lead to a nucleophilic attack on the mentioned flavonol, which induced the addition of 1,4 nucleophilic on the double bound to form an enol function. These observations could be very useful for investigating the mechanism of action, to gain better understanding of the strongest effect of KAE. The contribution of electronic and steric effects of the hydroxyl substitution on the B-ring was also investigated. The OH group in 4′ position in flavonols showed the highest influence and inhibitory potency against rat intestinal α-glucosidase activity that was explained by a hydrogen bridge bond (H-bond) between the 4′-OH and the enzyme [49]. For CpLIP2, the binding energy estimated using AutoDock Vina was −9.1, −8.6, −8.6, −8.1 and −7.8 kcal·mol^−1^ for GAL, KAE, QUE, MYR and THL, respectively. For each flavanol, the best pose corresponded to a binding site adjacent to W294 (Figure 11). THL had the lowest estimated binding energy by comparison to flavonols, which might explain why THL, despite its higher affinity for the catalytic site of the enzyme, is less competitive than flavonols on the accessible protein surface area when they are added together in the reaction medium.

### 3.2. Hydroxylation Degree of Flavonols Is Essential in CpLIP2 Lipase Inhibitory Mechanisms

As previously shown, OH groups of the flavonol B-ring play a crucial role in inhibiting the CpLIP2 lipase activity through specific hydrophobic or ionization interactions, probably close to the catalytic site of the enzyme. Indeed, GAL never resulted in more than 20% inhibition even after 30 min of pre-incubation, whereas with KAE the inhibition reached 90% at the highest concentrations. This effect seems to be mainly due to hydroxylation at C-4′ position of the B-ring of the flavonol skeleton. Indeed, when the number of hydroxy groups increased (two or three), the inhibition capacity was substantially reduced for QUE and MYR (by 2 or 3). We saw that the inhibition caused by flavonols does not correlate simply either with their hydrophobicity or with their degree of hydroxylation. Indeed, log_10_ P of partition between octanol and water phases were 3.5, 2.9, 2.1 and 3.9, respectively for KAE, QUE, MYR and GAL [50]. If we consider the log_10_D, corresponding to log_10_ P of partition (octanol/water at pH 7.4) of these flavonols, taking into account their ionization state [51], GAL reached the lowest value (0.7). Whereas GAL could be excluded due to very low capacity to form ionic bounds, the relatively high levels of hydrophobicity of the other molecules associated with ionization ability could be suitable for protein-phenolic interaction at neutral pH and would be a good compromise for inhibition. But, how might we understand such effects of hydroxylation on the enzyme, particularly in relation to its catalytic site as already mentioned above? Work done on the human erythrocyte carrier governing the glucose efflux revealed very similar results concerning its inhibition by KAE, QUE and MYR at pH 6.5. KAE was the strongest inhibitor, but when the pH increased to 8.5, MYR showed the highest effect with an increase of inhibitory potency by 10-fold for all flavonols tested [52]. Flavonoids holding hydroxy groups are considered as weak acids when they are deprotonated at high values of pH, conferring high inhibition capacity that clearly depends on pH [52]. In addition, a tight relationship was shown between the number of hydroxy groups in the molecule and its reduction capacity (release of protons H^+^), which could indicate the presence of charged molecules according to pH [53].

Moreover, KAE among 28 flavonoids tested at pH 7.4 including QUE and MYR exhibited the highest inhibitory activity against porcine pancreatic lipase (PPL) using a 4-methyl-umbelliferone oleate as substrate via fluorometrically assay [54]. The inhibition of rabbit PMNs oxidative metabolism stimulated via FcγR and/or CR membrane receptors was negatively correlated to the degree of hydroxylation for the three flavonols tested [50]. By contrast, the results of Tadera et al. [55], demonstrated that the inhibitory effect of porcine pancreatic α-amylase and α-glucosidase by flavonols was increased when the number of hydroxy group on the B-ring increases, as follows: MYR > QUE > KAE. It was reported that MYR showed the strongest inhibitory effect on phosphatidylinositol 3-kinase (PI 3-Kinase), among GAL, KAE and QUE [19]. By contrast, GAL was reported as inhibitor of pancreatic lipase (PL) activity using in vitro assay with IC_50_ value of 48.2 mg/mL [56]. However, based on the chemical structure of flavonols, it has been reported that multiple structural features are necessary for a selective inhibition of phospholipases (sPLA), including in QUE: 5-OH, 3′-OH and 4′-OH, beside the double bond C2-C3, and the presence of carbonyl group on C-4 [57,58]. Indeed, the high selectivity and specificity of QUE was exhibited on group II sPLA2 but not on group I sPLA2 [59]. The importance of the hydroxy group on the C-3′ and C-4′ of B-ring was signaled, but this specificity remains unclear [17]. The inhibition of aldose reductase was emphasized by the hydroxylation on C3′ and C4′ positions of B-ring [18]. It was reported that baicalein, which has an additional OH group on C-6 compared to GAL, was a less efficient inhibitor against rat intestinal α-glucosidase activity. The additional OH on baicalein C-2′, C-3′ and C-5′ decreased the baicalein inhibitory effect, while only the 4′-OH has emphasized its potency [49]. We can postulate that the requirement of mono-, di- or tri-hydroxylation on flavonol’s B-ring, identified as potent inhibitor, depends on the enzymatic matrix and the conditions of experiment, including the ionization form of flavonoids.

With reference to the review of Cao et al. on the impact of flavonoid structure on enzyme inhibition [18], we supposed that the inhibition of CpLIP2 lipase was established by hydrophobic and hydrogen bonds, added to intramolecular bonds between the OH group on C-5 and the oxygen atom of the carbonyl group, stabilizing the flavonol properties. Such substitutions do not interfere directly with the lipase itself. The structural features of flavonols known to be efficient lipase inhibitors were summarized as follows: the presence of a double bound between C2 and C3 on the C-ring that maintains the coplanarity of A and C rings, the presence of a carbonyl group on C-4 position, the presence of a OH group on C-5, and the presence of a OH group on the 4’-position. When one element is absent, the inhibitory effect can be deeply affected as seen with GAL, which is not hydroxylated on the 4′-position. It has also been reported that the absence of OH groups decreased the inhibitory potency of the flavonoids and it was already demonstrated on different enzymes such as sPLA [17], angiotensin-converting enzyme [60], digestive enzymes as α-glucosidase, α-amylase and aldose reductases [18]. Docking analyses of QUE against PLA2 gave additional evidence that this flavonol could interact through its hydrophobic ring A and/or B; the binding can be stabilized by π-stacking interactions of its A-ring with F residues. In addition, it was established that hydrogen bonds with proteins through the hydroxy groups of B-ring, beside intramolecular hydrogen bonds between C(5)-hydroxy group and the C(4)-carbonyl oxygen, allows it to keep its position within the hydrophobic site [17].

### 3.3. Mechanisms of Molecular Interactions between CpLIP2 Lipase and Flavonols Investigated by Means of Fluorescence and Docking

When complexation between flavonol and protein occurs, the fluorescence of aromatic amino acids residues of CpLIP2 is potentially reduced by two competing mechanisms. In the first one, large fraction of excitation energy is dissipated through an additional non-radiative de-excitation mechanism of the non-fluorescent complex, resulting in static quenching. For the second one, a fraction of this energy can be absorbed by flavonol, which secondly fluoresces to longer wavelength in a FRET mechanism. We showed that three of the seven W residues (W350, W294, W51) possess high ASA values corresponding to residues surrounding by water molecules, while two W residues (W177, W379) have a with very low ASA, corresponding likely to buried ones. As previously seen, each W of the CpLIP2 does not contribute similarly in term of fluorescence emission. At low concentrations of quenchers, F_0_/F plots showed a good linearity, which may reflect the fluorescence quenching of the three most exposed W residues holding the highest ASA values, as well as the two semi-buried W. However, at higher concentrations the curves of Stern–Volmer plots deviate drastically from the linearity, which may be explained by the presence of two buried W giving rise to the remaining fluorescence.

Few authors have reported spectroscopic studies concerning lipase interactions, but our results remain in good accordance with the identified static quenching mechanism, which was previously determined between QUE and PL via hydrogen bonds and van der Waals forces [61]. In addition, the binding forces between PL and QUE (among other flavonoids) presented the strongest binding combined to the highest inhibitory potency, mainly explained by its polar interactions due to its size and stringency of structure [62]. It was reported that F, L, isoleucine (I) and W residues play a major role in the dynamic of binding between proteins and flavonoid ligands according to: (i) flavonoids - amino acids (AA) binding energies, (ii) AA hydrophobicity, and (iii) the high number of AA involved in the binding sites of proteins [63].

### 3.4. Relationship between Binding Sites Number and Factors of W Accessibility to Flavonols 

Interestingly, this study showed that the level of inhibition of CpLIP2 lipase activity by flavonols having at least 1-OH group in their B-ring, correlated strongly with the quenching effect mainly explained by flavonol binding. Such a correlation has already been reported [41] between alpha-amylase and tea polyphenols. Indeed, the indole group of W side chain can contribute to different non-covalent interactions with other chemical species. It also participates in enzymatic catalysis and electron transfer [64]. Aromatic groups contain delocalized π electrons, which can interact with similar electrons in other aromatic groups, as well as with positively charged groups. The aromatic amino acids play an important role by forming closed scaffolds within proteins, especially binding sites for ligands and substrates [64]. The cation-π is one of the important non–covalent bounds in biological recognition, which is stronger than hydrogen bonds, when aromatic side chains are F, Y or W [65,66]. These authors reviewed the remarkable role of W residues on the cation-π with the ligand binding to numerous enzymes. Thus, W accessibility to quencher has a key role in the binding process of flavonols to lipase. Consequently, CpLIP2 lipase fluorescence quenching by flavonols depends on the three exposed W residues to the solvent. Thus, it was relevant to establish a tight relationship between the accessible surface area of W residues (ASA) and the factor of accessibility to quencher (*f_a_*). Such a relationship has not been discussed in previous studies, but warrants further investigations.

In conclusion, the interactions between CpLIP2 and the four flavonols or THL were studied successfully using enzymatic and quenching of fluorescence methods in parallel. CpLIP2 lipase was strongly inhibited by THL and KAE, less strongly by QUE and MYR, and weakly by GAL. One of the current challenges is to understand the mode of interactions between those flavonols and the CpLIP2 lipase, for identifying news drugs through relevant screening methods. For further investigations, two complementary approaches can be proposed: (i) an assay of competitive displacement [67] using the probe 8-analino-1-nephtalene sulfonic acid (ANS) could highlight the contrasted behavior between MYR and KAE and could allow to clarify which type of interactions (hydrophobic protein surface or electrostatic forces) occurs. (ii) Isothermal titration calorimetry (ITC) would be a promising method providing direct thermodynamic parameters for better understanding complexion between MYR and CpLIP2 lipase [68].

Our data showed that the 4′-position of the hydroxy group plays a crucial role in conferring the strongest inhibitory effect, while an additional OH on C-2′, C-3′ has a negative effect on the velocity of inhibition and the quenching of W fluorescence in the case of QUE and MYR. As already seen (Figure 8 and the corresponding text), the fluorescence study reveals that the binding of flavonols to CpLIP2 lipase depends on accessibility of its 7 W residues. However, the hydrophobic interactions seem to be essential for complexation and are probably responsible for enzymatic inhibition, as long as the flavonols remain in a protonated form. That is mainly determined by the pH of aqueous reactional medium according to the Pk_a1_ of ligands. Additionally, GAL had always the lowest effect. The correlations between the quenching of W fluorescence parameters and the inhibition rates of CpLIP2 activities were determined in order to explore the use of fluorescence emitted by proteins for future innovative inhibitory screening methods.

## 4. Materials and Methods

### 4.1. Chemicals and Reagents

Orlistat, i.e., tetrahydrolipstatin (THL, Pharmaceutical Secondary Standard), KAE (kaempferol, purity ≥97% by HPLC), QUE (quercetin, purity ≥95% by HPLC), MYR (myricetin, purity ≥96% by HPLC) and GAL (galangin, purity ~95% by HPLC) were purchased from Sigma-Aldrich (Saint-Quentin, Fallavier-France). Chemical structures are shown in Figure 1a,b.

### 4.2. Production of Recombinant CpLIP2 Lipase

The recombinant CpLIP2 lipase was produced by fed-batch cultivation of a transformed strain of *Komagataella (Pichia) pastoris* X-33 in a 5 L bioreactor as previously described [28].

Protein concentrations were determined by the Bradford method [69] using pure, lyophilized CpLIP2 as standard. Protein concentrations are therefore given in mg equivalent CpLIP2 protein per mL. The 3D structure of the protein has been described in [70].

### 4.3. Enzyme Activity Assay

Standard CpLIP2 hydrolytic activity measurement. Hydrolysis activities were determined by measuring the initial rates of fatty acid and esters production (µmol·min^−1^·mL^−1^), using ethyl oleate (C18:1, EE) as substrates. Specific activity is the total activity expressed in µmol·min^−1^·mL^−1^ in relation to protein concentration (mg·mL^−1^). Experiments were conducted as described by [28]. Hydrolysis of ethyl oleate (EO) was performed in aqueous emulsions at 30 °C. This emulsion consisted of 100 µmol·mL^−1^ EO emulsified by sonication (Branson Sonifier 250, 20 s, 200 watts, 145 µm amplitude) in an aqueous solution of 20 g·L^−1^ poly vinyl alcohol. The reactions of hydrolysis were prepared in glass test tube by adding 100 μL of ethyl oleate emulsion to 800 µL of sodium phosphate buffer 50 mM, pH 6.5; then the solutions were incubated in water bath at 30 °C for 5 min. The reaction was started by the addition of 100 µL of enzyme solution of CpLIP2 with final concentration 3 µg·mL^−1^ with activity 2000 U/mL.

The enzyme solution was pre-incubated at 25 °C, with continuous stirring at 350 rpm. Pre-incubation was conducted with 1980 µL CpLIP2 lipase diluted in sodium NaH_2_PO_4_/Na_2_HPO_4_ phosphate buffer (50 mM, pH 6.5) containing 20 µL absolute EtOH at 2 different times (1 and 30 min). This pre-incubation phase was conducted in closed vials placed during 2 h on a Radleys StarFish Multi-Experiment Workstation. The reaction was conducted for 15 min at 30 °C, and then stopped by adding 950 µL of a mixture of ethanol/sulphuric acid (100:0.8, *v/v*). After the addition of 50 µL of internal standards (ethanolic solution of pentadecanoic acid and its ethyl and methyl esters, 1 µmol each), lipids were extracted from the mixture with 1 mL of hexane. Samples were then prepared for analyzing esters and free fatty acids by gas chromatography (GC) as follows. Hexane extract (200 µL) was reacted with 25 µL pyridine and 25 µL *N*-methyl-tri (methylsilyl)-trifluoroacetamide (MSTFA) as silylating agent. Mixtures were incubated in water bath at 50 °C for 20 min, and then samples were ready for analysis. Reaction rates were determined by the analysis of fat composition by capillary gas chromatography after 15 min reaction. It was checked that less than 30% of the total substrate was converted at the end of the 15 min reaction [71].

A Shimadzu GC 2010 plus gas chromatograph equipped with a flame ionization detector, an automatic sampler (injected volume 0.1 μL) and a split/splitless injector was used for analysis. The capillary column was a DB-5ht (15 m × 0.25 mm, Phenomenex, Le Pecq, France). The helium carrier flow was 1 mL min^−1^and the split ratio was 1:50. Temperature conditions were, for reactions with individual substrates: injector 280 °C, detector 290 °C, oven 200–225 °C at 10 °C min^−1^ then to 260 °C at 35 °C·min^−1^ for ethyl ester (EE) and fatty acid (FA) of C18:1. Calibration curves were realized using emulsions of mixtures of FA and monoesters prepared according to the same protocol, without enzyme and alcohol.

### 4.4. Lipase Inhibition Assay and the Determination of CpLIP2 Inhibitory Rate 

In order to measure the residual lipase activity in the presence of potential inhibitors, the previous method was conducted in the presence and absence of the inhibitors [72]. For each potential inhibitor, the lipase-catalyzed rate of hydrolysis was evaluated after a pre-incubation period between the lipase and the potential inhibitors, which were solubilized in absolute ethanol at 1% of final concentration. The preincubation was performed at 25 °C with continuous stirring at 350 rpm with different concentration of inhibitors (14, 27.5, 55 µM) which represented, respectively 25, 50, 100-fold the final lipase concentration (0.55 µM). Five different times of pre-incubation were tested (1, 30, 60, 90 and 120 min), but only the results for 1 and 30 min have been shown, covering more than 80% of the inhibition rates. Systematically, the enzymatic activities were measured without inhibitor but with the same ethanol concentration for each experiment in the same conditions. Lipase inhibition was calculated from the residual activity in the presence of the compound under assay with respect to that of untreated samples (without inhibitor but prepared and analyzed under the same conditions than the inhibitor treated samples, and including the inhibitor solvent, ethanol absolute, to take into consideration the effect of each solvent in CpLIP2 activity). Inhibition rate was calculated by the following Equation (13):

Inhibition rate = ((A−B)/A) × 100(13)

where A was the quantity of the reaction product in the absence of inhibitor, and B was that in the presence of inhibitor [73,74].

### 4.5. Fluorescence Spectroscopy Measurement

#### 4.5.1. Apparatus

Fluorescence spectra and UV–vis spectra were recorded on microplate spectrofluorimeter model 6005271-Multimode Plate Reader (PerkinElmer, Waltham, MA, USA) conducted with Enspire Manager Program. The pH measurements were carried out on a pHmeter-pH3110 (Model WTW 82362, GmbH, Weilheim, Germany). The measurements of UV–Vis absorption and fluorescence were recorded using microplates 96 wells UV-star transparent or black with transparent bottom (Greiner bio-one GmbH, Frickenhausen, Germany).

#### 4.5.2. Sample Preparation and Well Constituent

To fill up the volume of 300 µL by well, 190 µL of sodium phosphate buffer solution (75 mM, pH 7.0) was added to each well to obtain final concentration at 50 mM. Then, 100 µL of ethanol absolute–water ultrapure (2:8, *v/v*) solution was added to obtain a final amount of ethanol (0.10 µmol) in the well of 300 µL. This concentration of ethanol did not affect the fluorescence spectrum of CpLIP2 lipase and was used to solubilize all the flavonols tested, whatever their high or low polarity, for quenching assays. The final concentration of ethanol during the experiments never exceeded 0.07% (*v/v*). The last step was to add 10 µL of lipase after to have optimized the final concentration of CpLIP2 equal to 2.5 µM for all our assays, giving the optimal fluorescence intensity for all measurements. The measurement of intrinsic fluorescence started after mechanical shaking for 30 s inside the multi-plate reader.

#### 4.5.3. Determination of Excitation and Emission Wavelengths

As mentioned above, the fluorescence of proteins comes from (W), (Y) and phenylalanine (F) residues, and CpLIP2 lipase possess 21 F; 22 Y; 7 W residues in its sequence. In this study, a new fluorescence measurement method was performed in buffered solution with a final concentration of ethanol less than 0.07% (*v/v*). The excitation and emission wavelengths for CpLIP2 lipase, the number of flashes (100), the high distance for measurement (13 mm) within each well, have been optimized. At 350 nm of emission, the excitation spectrum was chosen in order to avoid the emission peak of Y residues, which occurs at 305 nm [37]. Excitation spectrum of CpLIP2 lipase were recorded between 230 and 300 nm at the wavelength emission 350 nm, with 1 nm increment after an incubation of 5 min at 30 °C with stirring. Finally, the 285 nm as excitation wavelength (Figure S1, Supplementary Materials) was used to excite in priority the W residues which are the main responsible for the intrinsic fluorescence emission at 350 nm and providing an important tool to characterize its features [37]. Emission spectra of CpLIP2 lipase were recorded between 305 and 500 nm after an excitation at 285 nm, with 1 nm increment after incubation of 5 min at 30°C with stirring of the solution (Figure S1, Supplementary Materials).

#### 4.5.4. Effect of Ionic Strength and Temperature on Fluorescence Intensity 

In order to eliminate the aggregation phenomena and the instability of CpLIP2 lipase observed during 60 min of measurement of CpLIP2 fluorescence kinetic without NaCl, we examined the effect of ionic strength on the fluorescence emission intensity of CpLIP2 for final concentration (2.5 µM) by adding NaCl at increasing concentration ranges from 10 to 1000 mM (Figure S2, Supplementary Materials). For this experiment, 80 µL of ultra-pure water was replaced by adding 80 µL of saline solution. NaCl final concentrations that have been tested correspond to 0, 200, 300, 400, 600, 800, 1000 mmol·L^−1^. The most suitable concentration was determined at 300 mM NaCl, stabilizing the fluorescence, probably linked to the phenomenon of protein aggregation (Figure S2, Supplementary Materials). Furthermore, an increasing of fluorescence intensity was observed by adding 300 mM NaCl, which indicates a possible salting-out of the protein due to dehydration by addition of ions [75]. In addition, an increasing of CpLIP2 fluorescence intensity was recorded in presence of NaCl that indicates an interaction of salt ions with the lipase and a significant change in the aggregation on the ionic solution. A decreasing of fluorescence intensity of CpLIP2 lipase was observed when the temperature increased from 25 °C to 55 °C as shown in Figure S4 (Supplementary Materials).

### 4.6. Fluorescence Quenching

In order to reduce the effect of re-absorption and to remove the inner filter effects due to UV absorption, the whole fluorescence data were corrected by absorption of exciting light and emitted light based on the following relationship Equation (14):

F_c_ = F_m_ × e ^(A1+A2)/2^ (14)

F_c_ and F_m_ are the corrected and measured fluorescence. A1 and A2 represent the absorbance of experimental solution determined at the excitation and emission, respectively at 285 nm and 350 nm.

#### 4.6.1. Flavonols–CpLIP2 Lipase Interaction

CpLIP2 Lipase, THL and flavonols UV absorption spectra were performed under the same conditions of fluorescence measurements. The samples were scanned in water/ethanol with the range of 230–500 nm. As mentioned above, a mixture of 300 µL was pipetted in each well. A stock solution of GAL, KAE, QUE or MYR of 375 µM was prepared by dissolving flavonols in ethanol absolute. A scale of 10 successive concentrations was prepared from the stock solution of flavonols to obtain final concentration ranges (0, 2.5, 5.0, 7.5, 10.0, 12.5, 15.0, 17.5, 20.0, 22.5 and 25 µM) and 300 mM NaCl. The volume of 100 µL of 10 dilutions of different ligands were pipetted onto the plate. A solution of water/ethanol–NaCl saline solution 1.125 M (2:8, *v/v*) was added to wells including blank (Buffer) and positive control (lipase without ligand) to complete the volume if the ligand is absent. The measurements were performed at different temperatures (25, 35, 45 °C). Emission spectra of CpLIP2 lipase were collected between 305 and 500 nm with 1 nm increment after incubation of 30 min at 25 °C with stirring of the solution under excitation at 285 nm. Emission spectra of CpLIP2 lipase were recorded in the absence or presence of ligands.

#### 4.6.2. THL–CpLIP2 Lipase Interaction

As mentioned above, a mixture of 300 µL was pipetted in each well. 190 µL sodium phosphate buffer 50 mM, pH 7.0 with 100 µL of NaCl: EtOH (8:2, *v/v*) solution. 10 µL of native or denatured lipases at final concentration 2.5 µM were incubated for 50 min at 25 °C with 50 µM THL by adding 20-fold molar excess of CpLIP2 solubilized in ethanol absolute. In parallel, native and denatured lipases without THL were incubated in the same conditions. Denaturation of lipase was done by heating in water bath at 100 °C for 20 min.

#### 4.6.3. Quenching of CpLIP2 Fluorescence by KAE in the Presence of THL

Native CpLIP2 2.5 µmol·L^−1^ was pre-incubated for 30 min at 25 °C with increasing concentrations of THL to get inhibited CpLIP2. A scale of 10 successive concentrations was prepared from the stock solution of THL to obtain final concentration ranges (0, 2.25, 4.50, 6.75, 9.00, 11.25, 13.50, 15.75, 18.00, 20.25, 22.50 µmol L^−1^). The volume of 10 µL of 10 different THL dilutions were pipetted onto the well containing previously 190 µL of sodium phosphate buffer 75 mM, pH 7.0, and 80 µL of NaCl solution 1.125 M was added to obtain 300 mM NaCl. Measurement of fluorescence was effected at three temperatures (25, 35, 45 °C) for inhibited lipase by THL in the absence and the presence of KAE with fixed final concentrations 12.5 µM. 10 µL of KAE stock solution (375 µM) was added to the mixture to obtain final concentration 12.5 µM. The fluorescence emission intensity was recorded at 285 nm as excitation wavelength and 350 nm as emission wavelength.

### 4.7. Molecular Docking Analyses

3D model of CpLIP2 was previously illustrated [70]. The 3D structures of THL and the four flavonols tested, was drawn using Marvin Sketch (ChemAxon, Ltd, Budapest, Hungary). In silico molecular docking of the CpLIP2 inhibitors was performed using the AutoDock Vina program within UCSF Chimera (University of California, San Francisco, CA, USA) [76], that was also used for graphical representations. Solvent accessible surface areas (ASA) of CpLIP2 amino acids were computed using the MSMS package [77] within UCSF Chimera, using a probe radius of 1.4 Å and a vertex density of 2.

## Figures and Tables

**Table 1:** Biophysical parameters of molecular complexes formed between CpLIP2 lipase and flavonols (GAL, galangin; KAE, kaempferol; QUE, quercetin; MYR, myricetin) determined from Stern–Volmer equations at different temperatures (298, 308, 318, 328 K). The Stern–Volmer quenching constant (K_SV_), bimolecular quenching constant (K_q_), the fraction of accessible W residues to the quencher (*f_a_*), dissociation constant (K_d_), Stern–Volmer quenching constant of the exposed W residues (K_SV accessible_).

Equations		$\frac{F_{0}}{F}=1+K_{sv}\left[Q\right]=1+K_{q}\tau_{0}$			$\frac{F_{0}}{F_{0}-F}=K_{d}\frac{1}{\left[Q\right]}+1$		$\frac{F_{0}}{F_{0}-F}=\frac{1}{f_{a}K_{SV}}\frac{1}{\left[Q\right]}+\frac{1}{f_{a}}$	
Flavonol	T (K)	K_SV_ × 10^4^ L·mol^−1^	^a^R	K_q_ × 10^12^ L.mol^−1^ s^−1^	K_d_ × 10^−5^ mol·L^−1^	^b^R	*f_a_*	K_SV (__accessible)_ × 10^5^ L·mol^−1^
	298	2.9 ± 0.1	0.91	18	0.8 ± 0.3	0.72	0.37	3.6
GAL**	308	2.4 ± 0.9	0.92	15	0.8 ± 0.4	0.69	0.34	3.6
	318	2.1 ± 0.8	0.91	13	1.1 ± 0.6	0.73	0.33	2.9
	328	1.5 ± 0.6	0.86	10	1.0 ± 0.6	0.80	0.25	4.2
KAE*	298	2.1 ± 0.2	0.92	13	1.6 ± 0.3	0.97	0.45	1.4
	308	1.9 ± 0.7	0.95	12	1.4 ± 0.5	0.97	0.43	1.7
	318	1.8 ± 0.7	0.95	11	1.6 ± 0.7	0.96	0.42	1.5
	328	1.6 ± 0.6	0.95	10	1.9 ± 0.9	0.90	0.34	1.5
QUE*	298	1.8 ± 0.1	0.94	11	2.7 ± 0.6	0.98	0.54	0.7
	308	1.6 ± 0.6	0.93	10	2.4 ± 1.0	0.98	0.52	0.8
	318	1.6 ± 0.6	0.95	10	2.5 ± 1.0	0.98	0.55	0.7
	328	2.0 ± 0.7	0.91	13	3.1 ± 1.3	0.97	0.69	0.5
MYR*	298	1.6 ± 0.1	0.92	10	1.0 ± 0.1	0.96	0.34	2.9
	308	1.3 ± 0.5	0.93	8.2	1.1 ± 0.4	0.96	0.32	2.8
	318	1.2 ± 0.4	0.92	7.7	1.0 ± 0.4	0.95	0.30	3.2
	328	1.4 ± 0.5	0.94	9.0	1.2 ± 0.7	0.94	0.33	2.5

^a^ R is the correlation coefficient for the K_SV_ values. ^b^ R is the correlation coefficient for the K_d_ values. K_q_ = Bimolecular quenching constant, K_SV_ = Stern–Volmer quenching constant. (*) Concentrations of flavonols (2.5–25 µM). (**) Concentrations of flavonols (2.5–17.5 µM).

**Table 2:** Biophysical parameters of molecular complexes formed between CpLIP2 lipase and flavonols (GAL, galangin; KAE, kaempferol; QUE, quercetin; MYR, myricetin) determined from Stern–Volmer equations at different temperatures (298, 308, 318, 328 K). Number of binding sites (n), association constant (K_a_), enthalpy changes (ΔH), entropy changes (ΔS), and free energy change (ΔG).

Equations		$\log_{10}\frac{F_{0}-F}{F}=n\log_{10}\left[Q\right]+\log_{10}K_{a}$			$LnK_{a}=-\frac{\Delta H}{RT}\;\frac{\Delta S}{R}\;\Delta G=\Delta H-T\Delta S$		
Flavonol	T (K)	K_a_ (L·mol^−1^)	n	^c^R	ΔH (kJ·mol^−1^)	ΔG (kJ·mol^−1^)	ΔS (kJ·mol^−1^·K^−1^)
GAL** (Without OH)	298	323 ± 8	0.58 ± 0.18	0.85	0.07	−84.6	0.28
	308	502 ± 10	0.63 ± 0.21	0.85		−87.5	
	318	616 ± 10	0.66 ± 0.21	0.82		−90.3	
	328	5817 ± 2	0.66 ± 0.04	0.81		−93.1	
KAE* (1 OH)	298	458 ± 2.	0.63 ± 0.07	0.97	0.02	−39.3	0.13
	308	476 ± 4	0.63 ± 0.07	0.98		−40.6	
	318	862 ± 3	0.69 ± 0.09	0.97		−41.9	
	328	1290 ± 2	0.75 ± 0.07	0.94		−43.3	
QUE* (2 OH)	298	2034 ± 2	0.78 ± 0.06	0.95	0.02	−42.3	0.14
	308	1824 ± 9	0.77 ± 0.05	0.96		−43.4	
	318	3041 ± 2	0.81 ± 0.07	0.95		−45.2	
	328	4616 ± 3	0.85 ± 0.11	0.96		−46.6	
MYR* (3 OH)	298	45.21 ± 1	0.43 ± 0.02	0.97	0.02	−26.2	0.10
	308	53.09 ± 2	0.46 ± 0.03	0.96		−27.0	
	318	44.79 ± 1	0.44 ± 0.02	0.96		−27.9	
	328	97.14 ± 2	0.51 ± 0.09	0.96		−28.8	

^c^R is the correlation coefficient for the K_a_ values. (*) Concentration range of flavonol (2.50–25.0 µM). (**) Concentration range of flavonol (2.50–17.50 µM). The data are represented as mean value (±) Standard deviations of 5–9 values.

**Table 3:** The distance parameters calculated between the binding sites of CpLIP2 lipase and flavonols according to the chemical conditions previously described. GAL, galangin; KAE, kaempferol; QUE, quercetin; MYR, myricetin.

Flavonols	J (nm^4^·cm^−1^·M^−1^)	R_0_ (Å)	E	r (Å)	r/R_0_
GAL	0.8 × 10^14^	23.6	0.24	28.5	1.2
KAE	10 × 10^14^	36.0	0.12	50.1	1.4
QUE	12 × 10^14^	36.9	0.06	58.7	1.6
MYR	15 × 10^14^	38.4	0.23	46.7	1.2

## Supplementary Materials

The Supplementary Materials are available online. Figure S1: (**a**) Fluorescence excitation spectra of native CpLIP2 lipase, (**b**) Fluorescence emission spectra of native CpLIP2 lipase, Figure S2: (**a**) Kinetic of fluorescence emission of CpLIP2 lipase according to different concentrations of NaCl, (**b**) Effect of ionic strength on the fluorescence intensity at different times (20, 30 and 40 min) of incubation with CpLIP2, Figure S3: Fluorescence emission spectra of native (N) and denatured (D) free or inhibited CpLIP2 lipase, Figure S4: Fluorescence emission of CpLIP2 lipase as a function of temperature.
