# Supplementary material for: Inhibition of CpLIP2 Lipase Hydrolytic Activity by Four Flavonols (Galangin, Kaempferol, Quercetin, Myricetin) Compared to Orlistat and Their Binding Mechanisms Studied by Quenching of Fluorescence
Source: Molecules. 2019 Aug 8;24(16):2888. doi: 10.3390/molecules24162888 (PMC6719172; doi:10.3390/molecules24162888)
Supplement: Supplementary file 1 [file molecules-24-02888-s001.pdf]

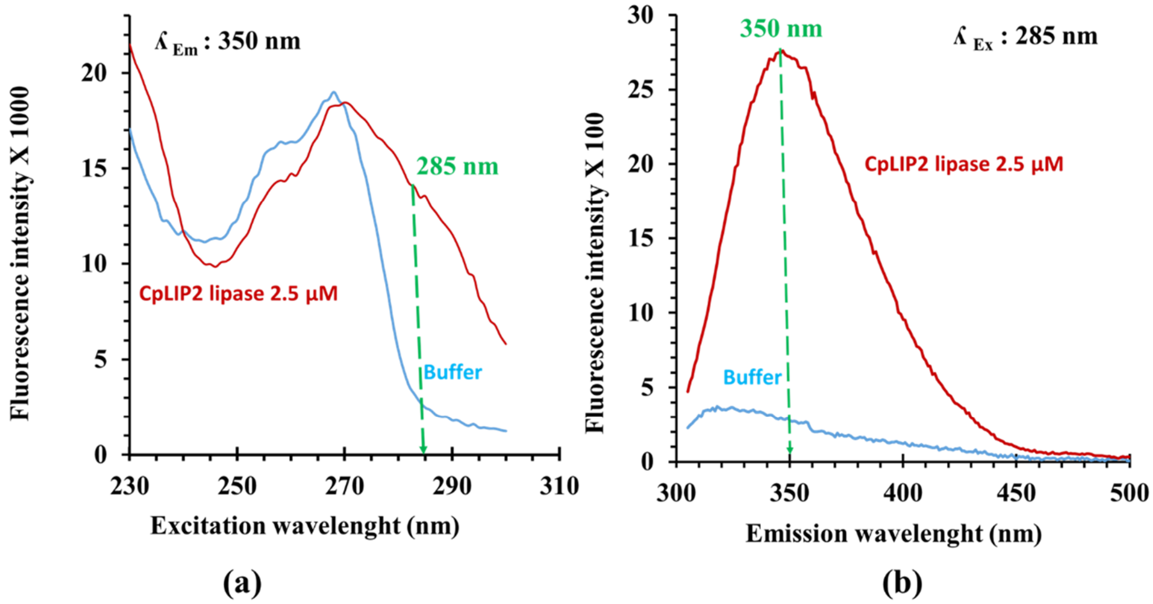

Figure S1. (a) Fluorescence excitation spectra of native CpLIP2 lipase (in red) and buffer (in blue) were recorded in the wavelength range of 230 - 300 nm. Slits set at 1 nm. The emission wavelength was 350 nm. (b) Fluorescence emission spectra of native CpLIP2 lipase (in red) and buffer (in blue) were recorded in the wavelength range of of 305 – 500 nm. Slits set at 1 nm. The excitation wavelength was x 285 nm. (a, b) in both measurements the concentration CpLIP2 was 2.5 x 10^{-6} mol L^{-1}. Measurements conducted at 303 K with sodium phosphate buffer (50 mM ; pH 7) without NaCl. W, tryptophan.

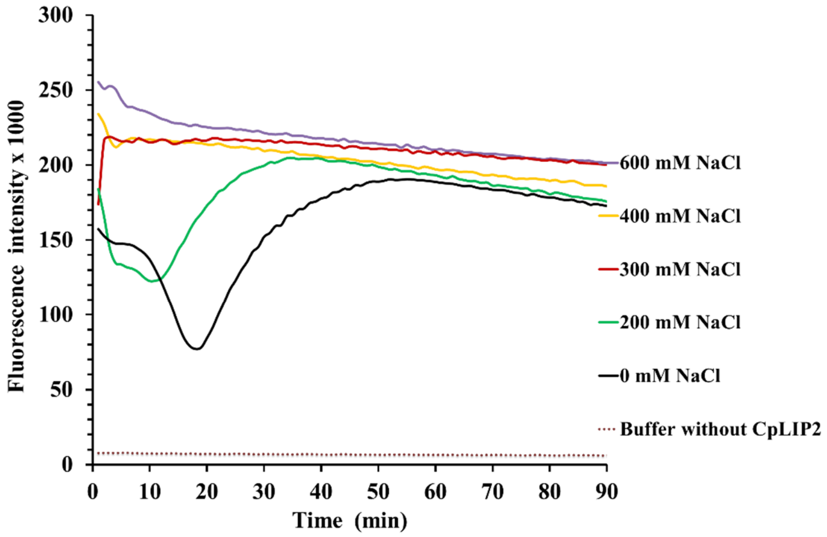

(a)

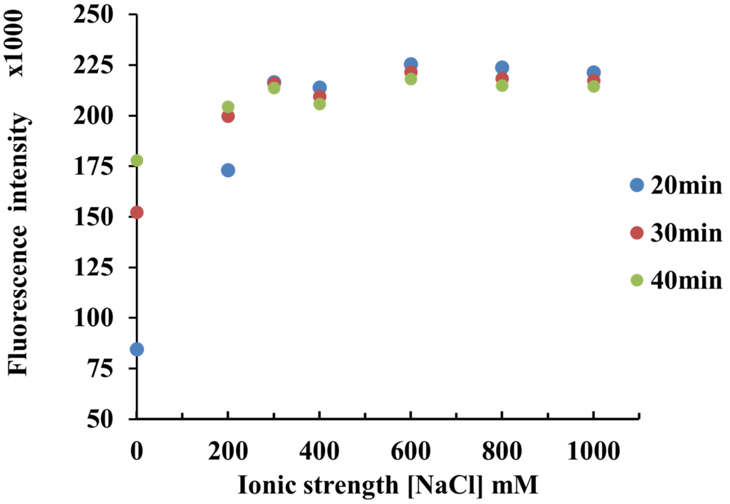

(b)

Figure S2. (a) Kinetic of fluorescence emission of CpLIP2 lipase according to different concentrations of NaCl. The concentration of CpLIP2 was 2.5 x 10^{-6} mol L^{-1}, NaCl final concentrations corresponding to 0, 200, 300, 400, 600, x 10^{-3} mol L^{-1} . Dotted line corresponds to basal fluoresence detected for sodium phosphate buffer (50 mM; pH 7) without CpLIP2. Measurement at (30°C = 293 K), pH : 7.0 ; EtOH 0.01%. The excitation wavelength was 285 nm, and the emission wavelength 350 nm. (b) Effect of ionic strength on the fluorescence intensity at different times (20, 30 and 40min) of incubation with CpLIP2 .

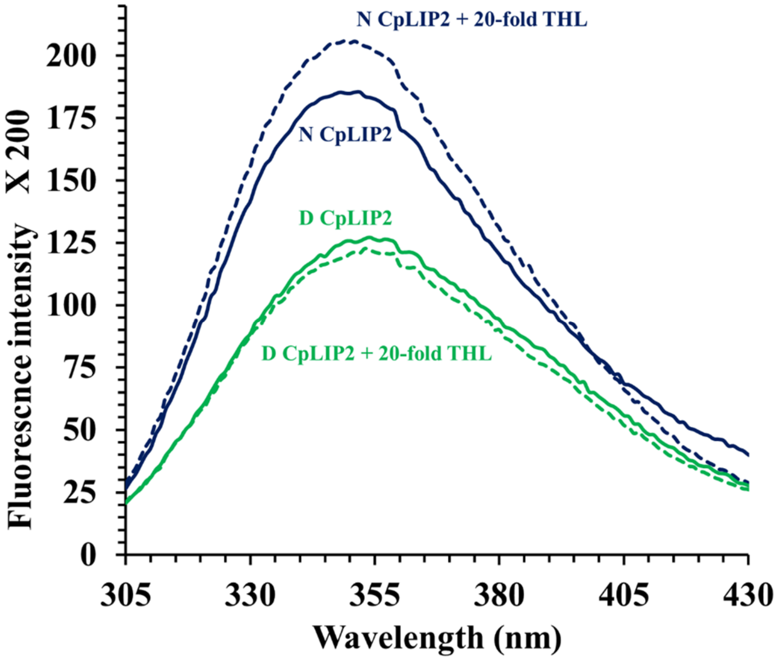

Figure S3. Fluorescence emission spectra of native (N) and denatured (D) free or inhibited CpLIP2 lipase. The concentration of native and denatured CpLIP2 was 2.5 x 10^{-6} mol L^{-1}. Inhibited CpLIP2 lipase was pre-incubated for 50 min at 25°C with 20 fold THL (final concentration 50 x 10^{-6} mol L^{-1}). Measurements were conducted at 293 K, in a sodium phosphate buffer (50 mM, pH 7) with 300 mM NaCl. The excitation wavelength was 285 nm, and the emission spectra was recorded in the wavelength range of 305 - 450 nm. Slits were set at 1 nm. The spectra correspond to: N, native free CpLIP2 or native CpLIP2 added with 20-fold molar ecxess orlistat (THL); (D) denatured free CpLIP2 or denatured CpLIP2 added with 20-fold molar ecxess orlistat (THL).

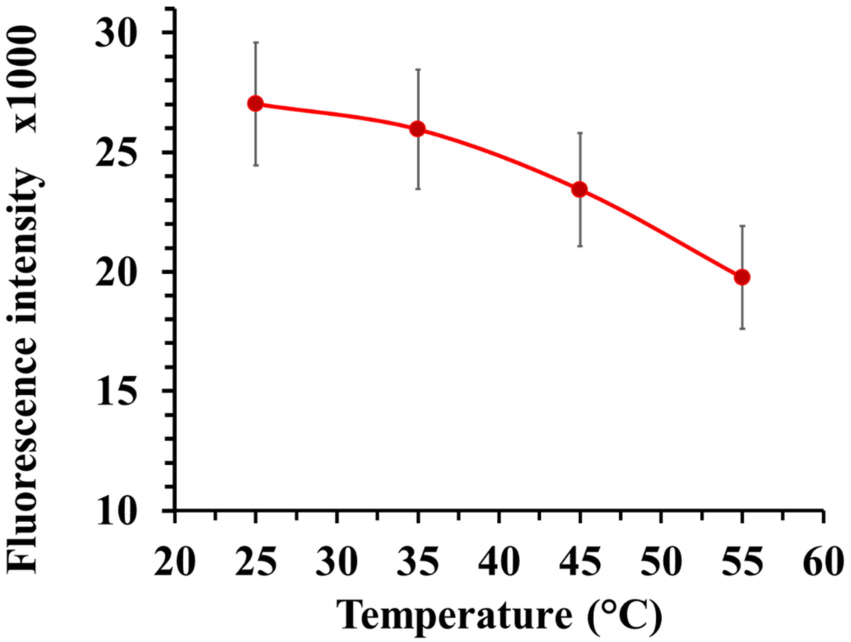

Figure S4. Fluorescence emission of CpLIP2 lipase as a function of temperature. The concentration of CpLIP2 was 2.5 µmol L^{-1}. Measurements were conducted in sodium phosphate buffer (50mM, pH 7.0) with 300 mM NaCl and absolute Ethanol 0.01%. The excitation wavelength was 285 nm, and the emission wavelength 350 nm. Data are presented as the mean ± SD (n= 30).
